# Sugar-induced cephalic-phase insulin release is mediated by a T1r2+T1r3-independent taste transduction pathway in mice

**DOI:** 10.1152/ajpregu.00056.2015

**Published:** 2015-07-08

**Authors:** John I. Glendinning, Sarah Stano, Marlena Holter, Tali Azenkot, Olivia Goldman, Robert F. Margolskee, Joseph R. Vasselli, Anthony Sclafani

**Affiliations:** ^1^Department of Biology, Barnard College, Columbia University, New York, New York;; ^2^Monell Chemical Senses Center, Philadelphia, Pennsylvania;; ^3^Obesity Research Center, Department of Medicine, Columbia University, New York, New York; and; ^4^Department of Psychology, Brooklyn College of City University of New York, Brooklyn, New York

**Keywords:** sweet taste, cephalic-phase insulin release, glucose tolerance, mice, T1r3

## Abstract

Sensory stimulation from foods elicits cephalic phase responses, which facilitate digestion and nutrient assimilation. One such response, cephalic-phase insulin release (CPIR), enhances glucose tolerance. Little is known about the chemosensory mechanisms that activate CPIR. We studied the contribution of the sweet taste receptor (T1r2+T1r3) to sugar-induced CPIR in C57BL/6 (B6) and T1r3 knockout (KO) mice. First, we measured insulin release and glucose tolerance following oral (i.e., normal ingestion) or intragastric (IG) administration of 2.8 M glucose. Both groups of mice exhibited a CPIR following oral but not IG administration, and this CPIR improved glucose tolerance. Second, we examined the specificity of CPIR. Both mouse groups exhibited a CPIR following oral administration of 1 M glucose and 1 M sucrose but not 1 M fructose or water alone. Third, we studied behavioral attraction to the same three sugar solutions in short-term acceptability tests. B6 mice licked more avidly for the sugar solutions than for water, whereas T1r3 KO mice licked no more for the sugar solutions than for water. Finally, we examined chorda tympani (CT) nerve responses to each of the sugars. Both mouse groups exhibited CT nerve responses to the sugars, although those of B6 mice were stronger. We propose that mice possess two taste transduction pathways for sugars. One mediates behavioral attraction to sugars and requires an intact T1r2+T1r3. The other mediates CPIR but does not require an intact T1r2+T1r3. If the latter taste transduction pathway exists in humans, it should provide opportunities for the development of new treatments for controlling blood sugar.

when a mammal ingests a sugar-sweetened beverage, it activates circuits in the gustatory neuraxis that perform three distinct functions: analyze its chemical composition (stimulus identification/discrimination), determine its acceptability (hedonic evaluation), and activate cephalic-phase responses (digestive preparation) (40, 41). While there is abundant evidence that the canonical sweet taste receptor T1r2+T1r3 is necessary for the identification and hedonic evaluation of sugar solutions (9, 33, 50, 57, 59), the question of whether T1r2+T1r3 is also necessary for digestive preparation is unresolved.

We focused on one aspect of digestive preparation: cephalic-phase insulin release (CPIR). It is elicited by pregastric contact with nutrients and enhances glucose tolerance (i.e., ability to maintain glucose homeostasis) in humans (22, 44) and rats (6, 18, 26, 37, 43, 47, 48). CPIR operates through the dorsal motor nucleus of the vagus (DMNX) in the medulla oblongata. Food-related sensory inputs (taste, trigeminal, and olfactory) activate parasympathetic neurons in the DMNX, which project to pancreatic beta-cells, release acetylcholine, and cause insulin release (5, 34, 44, 55). Sugars are potent elicitors of CPIR, but little is known about the nature of the underlying taste pathways. Furthermore, there is debate over which chemical stimuli elicit CPIR. For instance, in humans, there are reports that CPIR is elicited by sugars (55), sugars and artificial sweeteners (20), sugars but not artificial sweeteners (10), or neither (1, 45). In rats, one report indicated that CPIR is elicited by glucose alone (18), whereas others indicated that it is elicited by multiple sugars and artificial sweeteners (6, 35, 37, 47, 48).

Because sugars and artificial sweeteners are ligands of T1r2+T1r3 (25, 30, 31), they may be able to elicit a CPIR by activating the T1r2+T1r3-dependent taste signaling pathway (7). However, T1r2+T1r3-independent signaling pathways could also contribute. This latter possibility is based on comparisons between C57BL/6 wild-type (B6) and T1r3 knockout (KO) mice in the C57BL/6 background. For instance, the chorda tympani (CT) taste nerve of both mouse groups responds similarly to 0.5 M glucose (9). Furthermore, taste cells in both B6 and T1r3 KO mice release GLP-1 (an incretin hormone) in response to oral stimulation with glucose (21). Mice also express several potential T1r2+T1r3-independent signaling mechanisms for sugars in their taste cells, including a sodium-glucose cotransporter (SGLT1), several glucose transporters (GLUTs), and an ATP-gated K^+^ glucose sensor (27, 49, 56).

The present study had three goals. The first was to measure sugar-induced CPIR in B6 and T1r3 KO mice and assess its role in glucose tolerance. Prior studies have established that T1r2 KO and T1r3 KO mice display impaired glucose tolerance (12, 28, 29, 38), but normal insulin sensitivity (38). However, because these prior studies administered the glucose postorally (i.e., intragastrically or intraperitoneally), they did not address the specific contribution of orally expressed T1r3 to insulin release and glucose tolerance. The second goal was to determine the sugar specificity of CPIR in B6 and T1r3 KO mice. The third goal was to confirm prior reports of divergent behavioral attraction and CT nerve responses to sugars across both mouse groups.

## METHODS

### 

#### Animals and housing conditions.

The wild-type (i.e., C57BL/6J, or B6) mice were purchased from the Jackson Laboratories (Bar Harbor, ME). The T1r3 KO mice were derived from parental stock produced by homologous recombination in C57BL/6J (B6) embryonic stem cells (9) and maintained in a colony at Barnard College. The T1r3 KO mice were identical to the B6 mice in all respects, except that they did not express the *Tas1r3* gene, which codes for the T1r3 subunit of the T1r2+T1r3 sweet receptor. Approximately equal numbers of males and females from each group were tested in each experiment. We provide sample sizes in the figure legends.

All tests involved young (7–10 wk of age) mice that weighed between 19 and 27 g. Each mouse was tested only once in the study to avoid experiential confounds. The mice were maintained in a vivarium with controlled temperature and humidity and a 12:12-h light-dark cycle. They were housed individually in polycarbonate tub cages (27.5 × 17 × 12.5 cm) with Bed-O'Cobs bedding (Andersons, Maumee, OH) and Nestlet cotton pads (Ancare, Bellmore, NY). The mice had unlimited access to tap water and chow (5001; PMI Nutrition International, Brentwood, MO), except where noted otherwise (see below). According to the manufacturer, the chow diet contained four carbohydrates at the indicated concentrations (by weight): starch (31.9%), sucrose (3.7%), lactose (2.0%), fructose (0.4%), and glucose (0.2%). Mice obtained water from sipper spouts (with a 1.5-mm hole) attached to water bottles, which were placed on the wire cage top.

All animal procedures were approved by the Institutional Animal Care and Use Committee of Columbia University and conducted in accordance with the National Institutes of Health *Guidelines for the Care and Use of Laboratory Animals*.

#### Test solutions.

All chemicals were purchased from Sigma-Aldrich (St. Louis, MO) and were dissolved in deionized water. The solutions were all prepared on the day of testing and (unless stated otherwise) were presented at room temperature.

#### Phenotypic and genotypic screen.

Before testing was started, all mice were subjected to a two-bottle preference test with 10 mM saccharin vs. water. Previous studies demonstrated that B6 mice are strongly attracted to 10–38 mM saccharin and that T1r3 KO mice are either indifferent or mildly deterred by it (9, 15, 50, 57).

For each preference test, the mice were caged individually and provided chow ad libitum and two bottles. One bottle contained deionized water and the other 10 mM saccharin. The test was conducted over a 48-h period. For the first 24 h, the saccharin solution was placed on the left side of the cage; for the second 24 h, it was placed on the right side. Consumption from each bottle was measured after each 24-h period by weight. The results of this test confirmed that the B6 exhibit a strong (>90%) preference for the saccharin solution over water, while the KO mice exhibit no (<60%) preference.

The genotype of the B6 and T1r3 KO mice was confirmed by analyzing a random sample of the total test population (Transnetyx, Cordova, TN), using previously published primer information (9).

#### Blood glucose and plasma insulin measurements.

Before oral or intragastric (IG) administration of a sugar solution (see below), we water deprived mice for 23.5 h and food deprived them for 6 h. Water deprivation was necessary to motivate the mice to lick from the sipper tube during oral administration. Food deprivation was necessary to limit the quantity of food in the stomach of the mice during both oral and IG administration.

At the beginning of each trial (between 2:00 and 2:30 PM), we weighed the mice and then obtained a baseline tail blood sample (at 0 min). For blood glucose measurements, we collected five additional blood samples 5, 15, 30, 60, and 120 min after oral or IG administration. We collected a single drop of tail blood at each time point and measured plasma glucose with a hand-held glucometer (OneTouch Ultra, Milpitas, CA). For plasma insulin measurements, we collected four additional blood samples 5, 15, 30, and 60 min after the oral or IG administration. We collected a 30-μl sample of tail blood at each time point in an EDTA-coated capillary tube (Innovative Medical Technologies, Shawnee Mission, KS), centrifuged it for 3 min at 6,000 rpm, and decanted and stored the plasma (∼10 μl) at −80°C until analysis with an Ultra-Sensitive Mouse Insulin ELISA (Crystal Chem, Downers Grover, IL).

#### Oral glucose administration.

The mice were trained to lick from a sipper tube. All oral glucose administrations were conducted in a commercially available gustometer (Davis MS160-Mouse; DiLog Instruments), which permitted us to monitor the number of licks taken by each mouse.

To train the mice to drink in the gustometer, we subjected them to three training sessions. Immediately before each training session, we deprived each mouse of water for 22.5 h to motivate drinking. Afterwards, we placed the mouse in the gustometer; the training session began when it took its first lick and lasted for 30 min. On each training day, the mouse was permitted to drink water freely from a single stationary spout during the training session. Immediately afterwards, the mouse was returned to its home cage and given 1 h of ad libitum access to water. Then, it was water deprived for another 22.5 h. All mice adapted readily to the gustometer and the water-deprivation schedule and took between 250 and 500 licks per training session.

Immediately before an oral glucose administration, we deprived mice of water for 23.5 and food for 6 h. The administration procedure began once we obtained the baseline blood sample (henceforth, 0 min). Immediately afterwards, we put the mouse in the gustometer and gave it a maximum of 3 min to complete its weight-specific number of licks (see below). Once it did so, we closed the shutter (preventing any further licks) and transferred the mouse to a cage lacking food and water. The second blood sample was taken 5 min after the mouse took its first lick in the gustometer. If a mouse did not take the requisite number licks within 3 min, it was removed from the experiment.

#### IG glucose administration.

We subjected each mouse to the same water- and food-deprivation procedure as during oral glucose administration. Then, we secured the mouse by the scruff of its neck, gently inserted a curved feeding needle (Fine Science Tools, Foster City, CA) directly into its stomach (or the bottom of its esophagus), and injected a weight-specific volume of the test solution in <1 s.

### 

#### Insulin release and glucose tolerance following oral vs. IG glucose administration (experiment 1).

We subjected each B6 and T1r3 KO mouse to a single oral or IG administration of glucose solution. Subsequently, we made six blood glucose measurements (at 0, 5, 15, 30, 60, and 120 min) and five plasma insulin measurements (at 0, 5, 15, 30, and 60 min) per mouse.

We used a 2.8-M (50%) glucose solution because it enabled us to deliver the recommended body weight-specific dosage of glucose (i.e., 2 mg glucose/g mouse) (2) in an appropriate volume (e.g., 0.1 ml of 2.8 M glucose in a 25-g mouse). For the IG dosing, we used a 1-ml syringe attached to the feeding tube to ensure accurate mass-specific dosing. For oral dosing, we determined during pilot studies that B6 mice obtain, on average, 0.93 μl per lick from a 2.8-M glucose solution. Accordingly, a 25-g mouse would have to complete 107 licks in the gustometer to obtain the required 0.1 ml of 2.8 M glucose (i.e., 107 licks × 0.93 μl = 0.1 ml). This calculation generalized to an oral dosing regimen of 4.3 licks/g mouse.

#### What is the sugar specificity of the CPIR? (experiment 2).

Here, we examined changes in plasma insulin that occurred within 5 min of oral administration of one of three sugars (glucose, sucrose, and fructose) in B6 and T1r3 KO mice. We selected these sugars because they are all ligands of T1r2+T1r3 (25, 30) and stimulate high rates of intake in B6 mice (4, 13, 51). We used the same procedures as described above for measuring plasma insulin. Furthermore, the mice had to complete the same mass-specific number of licks (i.e., 4.3 licks/gram mouse) as in the previous experiment. Finally, the sugars were all presented at a 1-M concentration. We selected the 1-M concentration to accommodate the different molecular weights of the mono- and disaccharide sugars and because it approximates the concentration used by most other CPIR studies with rodents (11, 18, 47, 48).

We ran two negative control experiments. First, we asked whether the act of licking for water in a water- and food-restricted state was sufficient to induce insulin secretion in B6 and T1r3 KO mice. To this end, we used the same oral administration procedures described above, except that we offered deionized water to the experimental mice and no water to the control mice in the gustometer. Second, we asked whether the IG administration procedure (e.g., physical restraint and insertion of the feeding tube) would alter the time-dependent changes in plasma insulin in B6 mice. To this end, we repeated the oral administration procedure described above, using 2.8 M glucose as a stimulus. However, half of the mice were subjected to a sham gavage treatment immediately before oral administration, while the other mice were spared the gavage treatment before oral administration.

#### Are B6 and T1r3 KO mice differentially attracted to the 1-M sugar solutions? (experiment 3).

We used a no-choice two-bottle testing paradigm (14) to evaluate the oral acceptability of 1 M glucose, 1 M fructose, and 1 M sucrose to the B6 and T1r3 KO mice. We selected the 1-M concentration of each sugar because that is what was used in *experiment 2*.

Each test session lasted 20 min, during which time the mouse initiated a series of licking trials with two solutions: water or a sugar solution. It was provided access to a single solution during each trial. A trial began when the mouse took its first lick and ended 5-s later. We recorded both the number of licks the mouse emitted during each 5-s trial and the number of trials initiated. Because each trial was separated by a 7.5-s intertrial interval, a mouse could initiate up to 96 trials across the 20-min test session. To control for order effects within a test session, we treated the two test solutions (i.e., water and sugar solution) as a block and randomized (without replacement) their order of presentation within a block so that each solution was presented once before the next block began.

We subjected each mouse to three test sessions, each on separate days. During each test session, the mouse was offered a different pair of solutions, i.e., glucose/water, sucrose/water, or fructose/water. To control for order effects across test sessions, we tested each sugar/water pair in a counterbalanced manner across the mice. Therefore, for instance, during the first test session, approximately equal numbers of mice were tested with the glucose/water, sucrose/water, or fructose/water pair.

Before testing was started, each mouse received three training sessions with water in the gustometer. This served to familiarize the mice with the gustometer and train them to obtain fluid from the sipper tube. Each training session began when the mouse took its first lick and lasted 30 min. On training *day 1*, the mouse could drink freely from a single sipper tube throughout the session because the shutter was permanently open. On training *days 2* and *3*, the mouse could only drink from a sipper tube during sequential 5-s trials.

During training and testing, we used different restriction schedules to motivate mice to initiate large numbers of trials (16). During training, we water deprived the mice for 22.5 h before each session so as to motivate licking for water. Immediately afterwards, the mouse was returned to its home cage and was given 1 h of ad libitum access to water and food; then, it was water deprived for another 22.5 h. During testing, we food and water restricted the mice by giving them 1 g of laboratory chow (dustless precision 1 g food-pellets; BioServ) and 2 ml of water 23.5 h before each session. This latter deprivation schedule causes mice to lick avidly for sugar solutions but not plain water. Afterwards, the mouse was given a recovery day, during which it had food and water ad libitum.

#### CT responses to sugars (experiment 4).

The methodological details of the nerve recordings are described in detail elsewhere (13). In brief, the CT nerve innervates taste buds in the fungiform papillae, which occur primarily on the anterior portion of the tongue. We recorded from the CT nerve in the middle-ear cavity, while the mice were anesthetized with 1–5% isoflurane (Butler Schein, Albany, NY). During the surgery and recording procedure, the mice were maintained on a thermostat-controlled circulating-water heating pad set at 37°C (HTP-1500; Adroit Medical Systems, Loudon, TN). Neural responses were amplified 10,000× with an optically coupled isolated bio-amplifier (ISO-80; World Precision Instruments), passed through a band-pass filter (40 - 3,000 Hz), and then digitized (sampling rate = 2,000 samples/s), transformed (root mean square), and integrated (time constant = 1 s) (Biopac software, Goleta, CA).

We dissolved the chemical stimuli in an artificial saliva solution (32), consisting of deionized water plus NaCl (0.015 M), KCl (0.022 M), CaCl_2_ (0.003 M), and MgCl_2_ (0.0006 M). We used 0.25, 0.5, 1.0, and 2.0 M glucose and fructose; and 0.03, 0.1, 0.3, and 1 M sucrose. We used 0.1 M NH_4_Cl as a reference stimulus to be consistent with previous studies (9, 13).

We delivered the artificial saliva and tastant solutions to the anterior tongue (at a rate of 10 ml/min) with a continuous-flow system (VC-6 Perfusion Valve Control System; Warner Instruments, Hamden, CT). All solutions were kept at 35°C with an automatic temperature controller (Warner Instruments). The tongue was rinsed continuously with artificial saliva, both before and after each 20-s stimulation trial. To control for time-dependent changes in CT nerve responsiveness, we stimulated the tongue with the reference stimulus immediately (i.e., 40 s) before stimulating it with each of the sugar solutions.

The dependent measure was the relative response of the CT nerve to each sugar solution. We used Biopac software to determine the mean voltage during the 20 s immediately before chemical stimulation (= baseline response) and during chemical stimulation with the sugar solution (= excitatory response). Then, we measured the difference between the baseline and excitatory response (= absolute response). Finally, we divided the absolute response to a sugar solution by the mean absolute response to NH_4_Cl, yielding the relative response to each sugar solution.

#### Statistics.

In all experiments, we set the alpha-level at 0.05. We used IBM SPSS Statistics, v22 (www14.software.ibm.com) and Prism (http://www.graphpad.com) to analyze the data.

For the blood analysis, we used blood glucose concentration (in mg/dl) and plasma insulin concentration (in ng/ml). We analyzed the glucose and insulin data with mixed-model ANOVAs, using time and administration method as independent variables. Whenever the sphericity assumption was violated for the repeated-measures ANOVAs, we used the Greenhouse-Geisser correction to adjust the degrees of freedom (17). To determine whether there was a significant rise within 5 min of initiating licking, we compared the insulin samples collected at 0 and 5 min with a paired *t*-test.

For the lick data, we determined the mean number of licks emitted per trial for each of the test solutions. We used a paired *t*-test to compare the number of licks emitted/trial for the two test solutions during a test (e.g., 1 M glucose vs. water), separately for each mouse group and sugar. We also compared the total number of trials initiated (as a measure of ingestive responsiveness) across the two mouse groups during a given test (e.g., 1 M glucose vs. water), using an unpaired *t*-test.

For the CT nerve recordings, we analyzed the relative nerve responses with a two-way mixed-model ANOVA, separately for each sugar. We treated mouse group as a between factor and sugar concentration as a within factor. We also ran post hoc mouse group comparisons at each concentration, using Bonferroni's multiple comparisons test.

## RESULTS

### 

#### Insulin release and glucose tolerance following oral vs. IG glucose administration (experiment 1).

We examined the temporal dynamics of plasma insulin and blood glucose following oral (i.e., licking) vs. IG administration of a 2.8-M glucose solution in B6 and T1r3 KO mice.

**Fig. 1.**
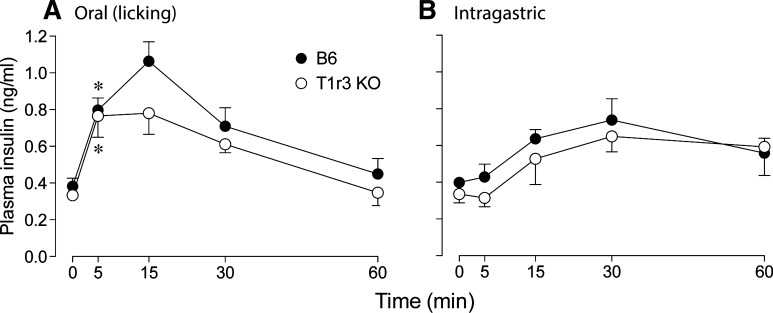
Plasma insulin levels in B6 and T1r3 knockout (KO) mice following oral (i.e., licking; *A*) or intragastric (*B*) administration of 2.8 M (50%) glucose (dosage = 2 mg/g mouse). *Insulin levels increased significantly (*P* < 0.01; one-sample *t*-test) above baseline within 5 min of the glucose challenge. Symbols represent means ± SE; *n* = 6 mice per mouse group and administration technique.

We compared changes plasma insulin levels over time across mouse groups, separately for mice that received oral (Fig. 1*A*) or IG (Fig. 1*B*) administration of the 2.8 M glucose solution. A mixed-model ANOVA revealed a significant main effect of time (*F*_4 84_ = 16.7, *P* < 0.001), reflecting the fact that plasma insulin increased and then decreased over the course of the 60-min test. Although the main effect of administration method was not significant (*F*_1,21_ = 3.5, *P* = 0.074), there was a significant interaction of time × administration method (*F*_4,84_ = 11.5, *P* < 0.001); this shows that the changes in plasma insulin over time differed across administration method. Indeed, one-sample *t*-tests revealed that plasma insulin in both B6 and T1r3 KO mice increased significantly within 5 min of the oral glucose challenge (in both cases, *P* < 0.009) but not within 5 min of the IG glucose challenge (in both cases, *P* > 0.55). This latter result indicates that the rapid elevation in plasma insulin following oral stimulation with glucose reflects a CPIR. Finally, the main effect of mouse group was not significant (*F*_1,21_ = 2.6, *P* > 0.12), indicating that T1r3 did not contribute to the dynamics of the insulin response.

**Fig. 2.**
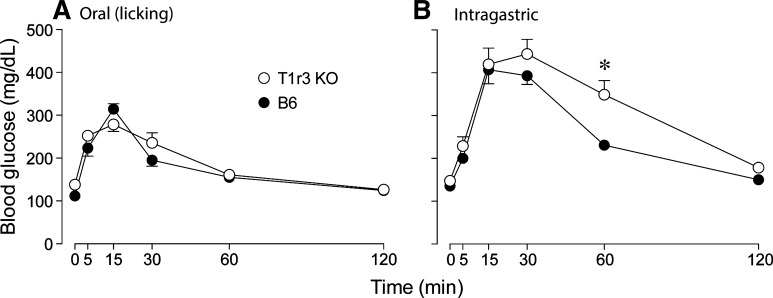
Glucose tolerance in B6 and T1r3 KO mice following oral (i.e., licking; *A*) or intragastric (*B*) administration of 2.8 M (50%) glucose (dosage = 2 mg/g). *Plasma glucose levels differed significantly (*P* < 0.05, unpaired *t*-test) between mouse groups. Symbols represent means ± SE; *n* = 6 mice per mouse group and administration technique.

We compared changes in blood glucose levels over time across mouse groups, separately for mice that received oral (Fig. 2*A*) or IG (Fig. 2*B*) administration of the 2.8-M glucose solution. The mixed-model ANOVA revealed a significant main effect of administration method (*F*_1,20_ = 34.6, *P* < 0.001) and time (*F*_5,100_ = 136.9, *P* < 0.001) but not of mouse group (*F*_1,20_ = 3.0, *P* = 0.1). These findings illustrate that plasma glucose peaked within 15–30 min of the glucose challenge and that mice from both mouse groups tolerated the glucose challenge significantly better following oral than IG administration. The significant interaction of time × administration method (*F*_5,100_ = 30.7, *P* < 0.001) reflects the fact that the rise in blood glucose persisted longer following IG administration, particularly in the T1r3 KO mice. Indeed, at 60 min, blood sugar was significantly higher in T1r3 KO than B6 mice (unpaired *t*-value = 3.34, df = 10, *P* < 0.008).

#### What is the sugar specificity of the CPIR? (experiment 2).

**Fig. 3.**
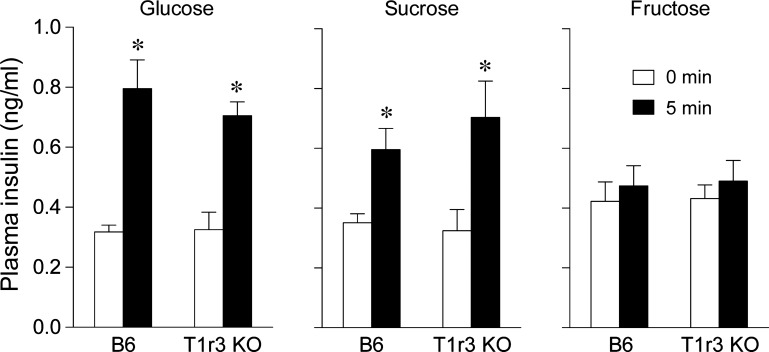
Plasma insulin levels at baseline (i.e., 0 min) and 5 min after initiating licking for one of three sugar solutions (1 M glucose, 1 M fructose, and 1 M sucrose) in B6 and T1r3 KO mice. Each mouse took 4.3 licks/g mouse from each solution. *Plasma insulin levels increased significantly (*P* < 0.05, paired *t*-test) above baseline within 5 min. Bars indicate means ± SE; *n* = 6–14 per sugar for B6 and T1r3 KO mice.

In Fig. 3, we show plasma insulin levels before (i.e., at 0 min) and 5 min after licking for 1 M glucose, 1 M sucrose, and 1 M fructose in both mouse groups. Plasma insulin levels were significantly higher 5 min after licking for the glucose and sucrose solutions in both B6 and T1r3 KO mice. This is revealed by a significant main effect of time (for both sugars, *P* < 0.0001) but not of mouse group (for both sugars, *P* > 0.6) or interaction of time × strain (for both sugars, *P* > 0.16). In contrast, plasma insulin levels did not increase significantly after licking for the fructose solution in either mouse group, as indicated by nonsignificant main effects of time (*F*_1,11_ = 3.1, *P* > 0.11) and mouse group (*F*_1,11_ < 0.1, *P* > 0.88) and a nonsignificant interaction of time × mouse group (*F*_1,11_ < 0.91, *P* > 0.47). Paired *t*-tests, conducted separately for each sugar and mouse group, confirmed a significant CPIR in response to 1 M glucose and 1 M sucrose (in all comparisons, *P* < 0.012) but not 1 M fructose (in all comparisons, *P* > 0.27).

**Fig. 4.**
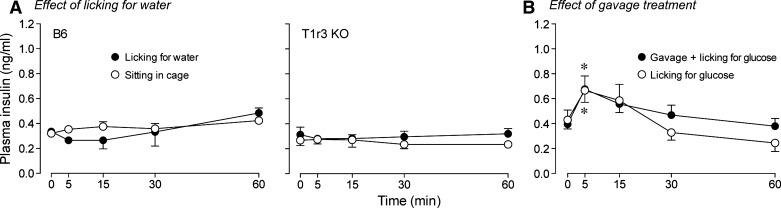
Negative control experiments to determine whether licking alone or the sham gavage treatment altered plasma insulin levels. *A*: plasma insulin levels in B6 and T1r3 KO mice after licking for water vs. sitting in the cage (*n* = 4–6 mice per mouse group and treatment group). *B*: plasma insulin levels in B6 mice after the sham gavage treatment + licking for 1 M glucose vs. licking for 1 M glucose alone (*n* = 6–8 mice per treatment group). *Insulin levels increased significantly between 0 and 5 min (*P* < 0.02; paired *t*-test).

We ran two negative control experiments. First, we asked whether licking for water alone elicited insulin release in B6 or T1r3 KO mice (Fig. 4*A*). A mixed-model ANOVA (performed separately on each mouse group) revealed no significant main effect of treatment (i.e., licking for water vs. sitting in cage) or time (in each case, *P* > 0.26) on plasma insulin levels. This shows that neither the act of licking nor the sensory input associated with ingesting water altered plasma insulin levels. Second, we asked whether the sham gavage treatment altered the time-dependent changes in plasma insulin levels after licking for 2.8 M glucose in B6 mice (Fig. 4*B*). The main effect of time was significant (*F*_4,48_ = 11.30, *P* < 0.0001) but that of the treatment (i.e., sham gavage + licking for glucose vs. licking for glucose) was not (*F*_1,12_ = 0.25, *P* > 0.61); likewise, the interaction of treatment × time was not significant (*F*_4,48_ = 1.0, *P* > 0.39). Plasma insulin levels increased significantly (*P* ≤ 0.02) between 0 and 5 min in mice from both treatment levels. The latter findings show that the sham gavage treatment itself did not alter the time-dependent changes in plasma insulin following the ingestion of glucose.

#### Are B6 and T1r3 KO mice differentially attracted to the 1-M sugar solutions? (experiment 3).

Previous studies have reported that T1r3 KO mice do not show appetitive licking for sucrose and glucose solutions in short-term lick tests (50, 57, 59). Here, we used a two-bottle acceptability test to confirm that T1r3 KO mice in our laboratory do not show appetitive licking for the sugar solutions used in the CPIR studies (i.e., 1 M glucose, 1 M sucrose, and 1 M fructose).

**Fig. 5.**
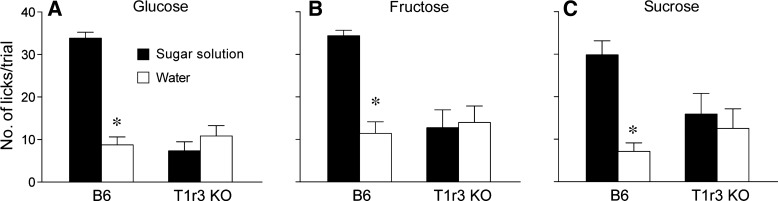
Licking by B6 and T1r3 KO mice for a sugar solution or water during 5-s trials. During each 30-min test session, mice were offered 2 sipper tubes serially. The tubes dispensed 1 M glucose or water (*A*), 1 M fructose or water (*B*), and 1 M sucrose or water (*C*). We compared the mean (±SE) number of licks per 5-s trial for the sugar solution vs. water, separately for each mouse group and sugar (*P < 0.05; paired *t*-test); *n* = 9 animals per mouse group; each mouse was tested with all 3 sugars in a counterbalanced design.

The B6 mice exhibited high rates of licking for the three sugar solutions (range: 30–35 licks/trial) but not for the water (range: 8–12 licks/trial) (Fig. 5). When we compared licks for each sugar solution vs. water, the paired *t*-values were all >6.3 (df = 8, *P* < 0.0001). In contrast, the T1r3 KO mice exhibited low and statistically indistinguishable rates of licking for each sugar solution and water (in all comparisons, paired *t*-values were <1.9, df = 8, *P* > 0.10).

There were also large mouse group differences in number of trials initiated. The B6 mice initiated significantly more trials per test session (range: 20–27) than the T1r3 KO mice during each of the sugar tests (range: 10–11). In all comparisons, unpaired *t*-values were >2.3 (df = 8, *P* < 0.035).

These results reveal that the B6 mice licked more avidly for the glucose, sucrose, and fructose solutions than for water during the short-term lick tests, whereas the T1r3 KO mice did not. In *experiment 2*, by contrast, both types of mice generated equally robust CPIRs in response to glucose and sucrose but not fructose. Thus the ability of the sugars to elicit CPIR in both mouse groups varied independently of their ability to stimulate licking.

#### Do B6 and T1r3 KO mice display CT nerve responses to sugars? (experiment 4).

We asked whether the CT nerve of the B6 and T1r3 KO mice generated significant responses to glucose, sucrose, and fructose. Prior studies reported that T1r3 KO mice (as compared with B6 mice) exhibit highly attenuated CT nerve responses to 0.5 M fructose (9), 0.1–1.0 M sucrose (59), and 0.5–0.6 M glucose in some (57) but not all (9) studies.

**Fig. 6.**
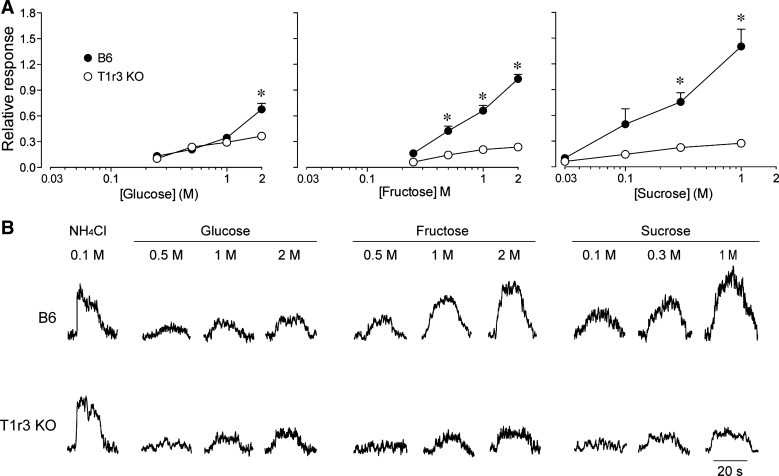
Chorda tympani (CT) nerve responses of B6 and T1r3 KO mice to lingual stimulation with various concentrations of glucose, fructose, and sucrose. *A*: relative responses reflect the ratio of the integrated response to a sugar solution divided by that to 0.1 M NH_4_Cl. We tested 10 B6 and 11 T1r3 KO mice with glucose; 11 B6 and 18 T1r3 KO mice with fructose; and 7 B6 and 7 T1r3 KO mice with sucrose. We compared the magnitude of response across mouse groups, separately for each concentration, using Bonferroni's multiple comparison tests. *Significant mouse group differences at a given concentration (*P* < 0.05). *B*: typical whole nerve integrated CT nerve responses to 0.1 M NH_4_Cl and different concentrations of glucose, fructose, and sucrose in B6 and T1r3 KO mice.

Figure 6 shows CT nerve responses of B6 and T1r3 KO mice to a range of glucose, fructose, and sucrose concentrations. For glucose, there were significant main effects of mouse group (*F*_1,19_ = 10.9, *P* < 0.004) and concentration (*F*_3,57_ = 78.0, *P* < 0.0001) and a significant interaction of mouse group × concentration (*F*_3,57_ = 15.1, *P* < 0.0001). These results reflect the fact that the magnitude of the CT nerve response was similar across mouse groups at concentrations ≤1 M, but that of B6 mice was significantly greater than that of T1r3 KO mice at the 2-M concentration. Finally, we conducted a repeated-measures ANOVA across glucose concentration, separately for each mouse group. These analyses confirmed that the relative response of the CT nerve to glucose increased significantly with concentration in both mouse groups (in each case, *P* < 0.0001).

For fructose, there were significant main effects of mouse group (*F*_1,27_ = 95.1, *P* < 0.0001) and concentration (*F*_3,81_ = 135.4, *P* < 0.0001) and a significant interaction of mouse group × concentration (*F*_3,81_ = 59.5, *P* < 0.0001). This reflects the fact that the CT nerve response of all three mouse groups increased with fructose concentration but that of B6 mice increased disproportionately at concentrations ≥0.5 M. We also ran a separate one-way repeated-measures ANOVAs across fructose concentration, separately for each mouse group. These analyses demonstrated that the CT nerve response to fructose increased significantly with concentration in both groups (in each group, *P* < 0.0001).

For sucrose, the main effects of mouse group (*F*_1,12_ = 24.0, *P* < 0.0005) and concentration (*F*_3,36_ = 26.9, *P* < 0.0001), and the interaction of mouse group × concentration (*F*_3,36_ = 14.0, *P* < 0.0001), were all significant. These results illustrate that the CT nerve response of both B6 and T1r3 KO mice increased with sucrose concentration but that of B6 mice increased at a significantly greater rate across all concentrations. For each mouse group, we ran separate one-way repeated-measures ANOVAs across sucrose concentration. These analyses revealed that the CT nerve response to sucrose increased significantly with concentration in both groups (in each case, *P* < 0.0001).

## DISCUSSION

The mouse has become an important model system for analyzing the molecular basis of glucose tolerance and insulin release. To identify the genes associated with glucose homeostasis, including *Tas1r2* and *Tas1r3*, investigators typically administer sugars via a postoral route (i.e., IG, intraperitoneally, or intravenously) to mice with gene deletions and then assess the functional consequences (2, 3, 12, 24, 28). While these postoral administration methods help identify the location of signaling or secretory cells involved in glucose homeostasis, they do not provide clinically relevant information because they bypass the oral stimulation associated with ingestion. Indeed, had we limited our studies to postoral stimulation, we would have grossly underestimated glucose tolerance in the mice. Accordingly, our results from mice, together with similar findings from humans (42), underline the importance of incorporating oral stimulation into glucose tolerance protocols.

When rats ingest glucose or sucrose, the latency for the sugar-induced insulin spike is typically <5 min, whereas that for the postabsorptive blood glucose spike is typically ≥5 min (37, 47). Because the CPIR occurred before the rise in blood glucose in these rat studies, the authors inferred that that the insulin spike was elicited by pregastric input and was thus a CPIR. We could not use a similar line of inference in this study because the insulin and glucose spikes both occurred in <5 min (Figs. 1 and 2). As a result, we focused on the fact that the insulin spike began in <5 min following oral administration but took >5 min to develop following IG administration. Indeed, there were no measureable changes in plasma insulin levels during the 5-min period immediately following IG glucose administration in the B6 and T1r3 KO mice. Based on these observations, we infer that the insulin spike following oral administration of glucose must have been elicited by oral sensory stimulation and was thus a CPIR.

The observation that it takes >5 min for insulin to reach detectable levels in the plasma following IG administration has been replicated in another laboratory (23). Such a long latency cannot be explained by *1*) a slow rate of glucose entry into the stomach because once the feeding tube was inserted, it took <1 s to inject the full dose of glucose; or *2*) stress associated with the IG gavage procedure because delivering a sham IG gavage, immediately before oral glucose administration, failed to alter plasma insulin levels over the next 60 min in the present study. Instead, we attribute the long latency of insulin release (following IG administration) to delays associated with the *1*) absorption of glucose from the intestine, *2*) transport of glucose (via the blood) to the pancreas, and *3*) a slow rate of insulin buildup in the plasma. Indeed, there is evidence that the latter process alone takes 2–3 min (8). On the other hand, we propose that oral administration of glucose caused more rapid insulin release by activating ascending taste pathways, which in turn stimulated parasympathetic neurons in the DMNX. Some of these parasympathetic neurons project to pancreatic beta-cells and thus could elicit insulin release directly (34).

Several observations support our inference that the CPIR was elicited by taste input. First, licking for water alone failed to elevate plasma insulin; this shows that the CPIR was not elicited by the act of licking for a fluid while in a water- and food-restricted state. Second, the CPIR was induced by 1 M glucose and 1 M sucrose but not 1 M fructose; this establishes that insulin release was not stimulated by an osmotic effect of the sugar solutions. Third, the CT nerve of both the B6 and T1r3 KO mice responded to glucose and sucrose solutions in the present study and elsewhere (9); this demonstrates that the sugar solutions generate a measurable and consistent peripheral taste response in both mouse groups.

There are two notable differences between the CPIR of mice and those of other mammals. In humans, the peak plasma insulin achieved during CPIR is ∼33% of that achieved during the postabsorptive insulin response (46). In B6 mice, the peak plasma insulin achieved during CPIR (measured at 5 min) is nearly 60% of the peak insulin levels achieved 15–30 min after oral administration (Fig. 1*A*). This interspecific discrepancy suggests that the relative magnitude of the CPIR is larger in mice, perhaps reflecting their higher mass-specific energy demands. Second, the CPIR is temporally distinct from the postabsorptive insulin peak in humans (46) and rats (26), resulting in a bimodal pattern of insulin secretion. We did not observe a similar bimodal pattern in mice. This may reflect the fact that we sampled plasma insulin too infrequently.

Our results show that T1r3 is not necessary for the generation of a normal CPIR. Indeed, 2.8 M glucose, 1 M glucose, and 1 M sucrose all elicited strong CPIRs in the B6 and T1r3 KO mice. The robust CPIR (and associated glucose tolerance) likely contributes to the ability of T1r3 KO mice to maintain normal fasting blood-glucose levels (38), daily chow intakes, and growth rates (52), relative to their wild-type counterparts. It is unlikely that the spared T1R2 subunit (either as a homodimer or in conjunction with a novel protein) mediated CPIR in the T1r3 KO mice. This is because sucrose, glucose, and fructose all elicit calcium release in heterologous reporter systems expressing T1r2+T1r3 but not in ones expressing T1r2 alone (25, 30) (P. Jiang, unpublished observations). Further, it seems improbable that a T1r2-mediated transduction pathway would generate a CPIR in response to sucrose and glucose but not fructose.

Other investigators have speculated about a T1r2+T1r3-independent taste transduction pathway for sugars in mice (9, 27, 49, 56). This study establishes that such a pathway exists and that it serves a critical metabolic function (i.e., eliciting CPIR and enhancing glucose tolerance) in mice. The fact that this pathway responds to glucose and sucrose, but not fructose, would appear incompatible with the hypothesized T1r2+T1r3-independent signaling mechanisms (i.e., SGLT1, GLUTs, and ATP-gated K^+^ channels), which are selective for glucose (27, 49, 56). However, there is some evidence that sucrase is expressed on the surface of taste cells (R. Margolskee, unpublished observations), and it is possible that the T1r2+T1r3-independent signaling pathway does not actually respond to sucrose. Instead, it may respond to the glucose liberated from sucrose by the catabolic action of oral sucrase.

In B6 and T1r3 KO mice, the relative magnitude of the CPIR to glucose, sucrose, and fructose varied independently of the relative acceptability of the same sugars. Likewise, in B6 mice, 1 M fructose elicited a robust CT nerve response but no CPIR. The most parsimonious explanation for these observations is that the taste system of mice contains two functionally distinct signaling pathways for sugars. One requires an intact T1r2+T1r3 to operate. It provides input to the gustatory cortex and ventral forebrain (40, 41) and mediates the attraction to sugars (e.g., glucose, sucrose, and fructose) and nonnutritive sweeteners. The other signaling pathway does not require an intact T1r2+T1r3 to operate. It provides inputs to the dorsal vagal complex (5, 34, 44, 55), responds to glucose and sucrose, and mediates CPIR and perhaps other cephalic-phase responses. Given that the T1r2+T1r3-independent pathway did not alter ingestive behavior, it follows that this pathway also did not generate a salient taste sensation. The existence of these two taste signaling pathways provides further support for the existence of multiple categories of taste processing, even within the same taste quality (40, 41).

Future studies need to determine the nature of the two hypothetical sugar transduction mechanisms in taste cells. At this point, we know that the majority of T1r3-expressing taste cells also express functional K_ATP_ channels (56). If K_ATP_ channels mediate CPIR, then we predict that they would generate a different output signal than the T1r2+T1r3-activated transduction mechanism. In support of this possibility, studies of immune cells show that the coexistence of multiple transduction mechanisms in the same cell creates the opportunity to generate a diversity of output signals (19).

### Perspectives and Significance

There are two important implications of the T1r2+T1r3-independent sugar pathway. One is that if this pathway exists in humans, then it should provide opportunities for the development of new treatments for controlling blood sugar. For instance, given the critical role of CPIR in glucose homeostasis, new therapies could be developed to modulate the strength of the CPIR. The second implication stems from the common assumption that mice with genetic deletions of T1r2, T1r3, or T1r3+T1r3 cannot taste sugars because they are not attracted to them during brief-access taste tests. Our results show that this assumption is incorrect. Even though the glucose and sucrose solutions failed to elicit appetitive licking in the so-called sugar-ageusic T1r3 KO mice, they nevertheless elicited a taste-mediated and physiologically significant CPIR.

It is likely that a portion of the CT nerve responses that we observed in the B6 and T1r3 KO mice reflected activity in the T1r2+T1r3-independent glucose/sucrose pathway. More work is needed to determine whether CPIR in mice is mediated exclusively by input from this nerve, as appears to be the case in rats (48), or whether input from the greater superficial petrosal and glossopharyngeal nerves also contributes. Given that glucose stimulates the release of GLP-1 from taste bud cells (21), it is also possible that taste cell-derived GLP-1 contributed to the CPIR.

In closing, we acknowledge two caveats in our study. We limited testing to relatively high concentrations of sugars, as in most prior rodent studies (11, 18, 47, 48). While future experiments need to examine lower concentrations, it is notable that these high concentrations have ecological relevance, e.g., glucose and sucrose together constitute between 8 and 44% of the total mass of many fruits (e.g., dates, rowal, jackfruit, mangos, apricots, and pineapple); and glucose constitutes >30% of the total mass of honey (http://ndb.nal.usda.gov/ndb/nutrients/index) (53). We also limited testing to mice that were naïve to sugar solutions, as we did not want our results to be confounded by potential conditioning effects (36, 39, 54, 58). Although the concentration of sucrose and glucose was low in the chow diet (3.7 and 0.2% by weight, respectively), it is possible that prior intake of this diet conditioned the observed sugar-induced CPIR. Future studies should determine whether dietary exposure to sugars alters the magnitude of sugar-induced CPIR.

## GRANTS

Funding was provided by a grant to Barnard College from the Howard Hughes Medical Institute.

## DISCLOSURES

No conflicts of interest, financial or otherwise, are declared by the author(s).

## AUTHOR CONTRIBUTIONS

Author contributions: J.I.G., J.R.V., and A.S. conception and design of research; J.I.G., S.S., M.H., T.A., and O.G. performed experiments; J.I.G. analyzed data; J.I.G., R.F.M., J.R.V., and A.S. interpreted results of experiments; J.I.G. prepared figures; J.I.G. drafted manuscript; J.I.G., R.F.M., J.R.V., and A.S. edited and revised manuscript; J.I.G., S.S., M.H., T.A., O.G., R.F.M., J.R.V., and A.S. approved final version of manuscript.
